# Advances against Aspergillosis and Mucormycosis

**DOI:** 10.3390/jof6040358

**Published:** 2020-12-11

**Authors:** David A. Stevens

**Affiliations:** 1 California Institute for Medical Research, San Jose, CA 95128, USA; 2 Division of Infectious Diseases and Geographic Medicine, Stanford University Medical School, Stanford, CA 94305, USA; stevens@stanford.edu

The 9th meeting of Advances Against Aspergillosis in beautiful Lugano, Switzerland clearly had the most drama of any of the previous meetings, exceeding even the 1st one, in San Francisco, when we, the Co-Organizers, weren’t sure that although we had a great educational idea, and had put together a great list of speakers and topics, we might have few attendees, and go bankrupt! (The story of the birth efforts in initiating these meetings is described, for the historical record [1].). More about the special circumstances of the 9th meeting, later.

Aspergillus and Mucorales are ubiquitous in nature, found on decaying matter, in soil and water, and have industrial and agricultural impacts, as well as medical [2]. While primarily opportunistic pathogens in man, in some species these fungi can be primary pathogens [2]. The *Aspergillus* field continues in a state of rapid advancement, including the publication of numerous postgenomic papers and substantial advances in translational, immunologic, epidemiologic and diagnostic research. The mucormycosis field is similarly advancing with novel studies in pathogenesis and diagnostic options. The launch of several antifungals in the recent past, development of more such drugs, and anticipated clinical trials of newer compounds with novel mechanisms of action is an exciting time for mycology. Pan-azole, and echinocandin *Aspergillus* resistance has emerged, in addition to the innate resistance that is a feature of many *Mucorales* clinical isolates, and requires unique approaches. Combination therapy remains an important area of interest for both aspergillosis and mucormycosis. Greatly increased awareness of allergic aspergillosis has opened new opportunities for both antifungal agents and immunotherapies. New molecular strategies for diagnosis continue to make progress, and recent guidelines offer increased diagnostic insight. There is, however, a continuing high death toll from invasive aspergillosis and mucormycosis, including patient groups not usually associated with these opportunistic infections [3]. The fundamental purposes of these international meetings continue to be to facilitate learning, teaching, and disseminating information, to gain perspective on the present status of disciplines (particularly outside those you are working on), and to enhance communication and engender collaborative relationships amongst clinicians, scientists, and industry to further advance the field. Beginning with the 8th conference, it was expanded to now include scientific sessions on mucormycosis pathogenesis, diagnosis, and treatment. As Advances Against Aspergillosis and Mucormycosis has become the leading global meeting for basic and clinical science regarding Aspergillus, its efforts form one of the foundations of the repository of knowledge about this pathogen. The excellent papers in this Journal of Fungi Supplement will meritoriously *add* to the 267 papers previously successfully published in 9 prior Supplements (one prior to the 1st meeting); those 267 papers comprised 2026 pages of full papers. The full papers are intended to be largely reviews. The abstracts of all accepted submissions are published in the program distributed at the time of the meeting (there have been 1594 such abstracts to date). Registration records from past meetings have indicated the attendees are infectious disease specialists, hematologists, oncologists, transplantation specialists, allergists, chest physicians, intensivists, other internists and pediatricians, clinical microbiologists, pharmacists, toxicologists, financial and pharmaceutical industry and hospital executives, governmental employees in healthcare and regulatory fields, veterinarians, environmental specialists, public health specialists, mycologists, immunologists, geneticists, epidemiologists, plant pathologists, other basic scientists, nurses, Fellows, Residents, and MD and PhD students. They come from dozens of countries.

All such diverse attendees will be interested in the subject matter of this Journal of Fungi Supplement. It is full of important compendia. There are papers detailing immunity to pulmonary mycoses, on *Aspergillus* products that trigger asthma, and on the role of fungi in unresponsive asthma. Other papers deal with *Aspergillus* regulatory metabolic networks, and on important non-*fumigatus Aspergillus* species. The molecular basis of *Pseudomonas-Aspergillus* competition is reviewed- so important in some human lungs, as is how volatile species are part of the intermicrobial competition. Regarding the *Mucorales*, there are reviews relevant to *Rhizopus* biochemical physiology, and to host defenses against these pathogens. The critical utility of animal models in understanding pathophysiology of fungal infections, and their treatment, is covered. In the realm of diagnostics, how siderophores can be useful in clinical detection of fungal pathogens is discussed, as is the latest in in vitro drug susceptibility methodology. Finally, how the International Society for Human and Animal Mycology is exerting collaborative efforts to advance the field is made evident. The open access policy of the Journal of Fungi makes all these important works so accessible to the entire scientific community, including those who did not have the benefit of the individual personal interactions, so important in scientific meetings, in Lugano.

I promised, above, a narrative about the drama: As chance would have it, the planes of two of the Co-Organizers, Bill Steinbach and I (and my wife, Julie, one of the scheduled attendees and volunteers for the meeting), coming from the US, and of Derry Green, in charge of the meeting planner operation (and her team, including her husband, Andy Green) coming from the UK, arrived in Zurich airport, just before the start of the meeting, within minutes of each other (while the 3rd Co-Organizer, David Denning, in the UK, had not left yet). The Zurich arrival group had arranged to rendezvous in the airport after their arrivals, and then connect by rail to Lugano, during which time we could chat leisurely about last-minute preparations, and the inevitable small last-minute issues that always come up. 

When we had our rendezvous, we were by that time gathering information that the coronavirus was beginning to close in on us. The epicenter outside of China at the time appeared now to be northern Italy, which, in a way, logistically, surrounds Lugano. The alternate route to Zurich, in order to get to Lugano, is Milan, which was the plan for many speakers and attendees. There was talk in the media that all of northern Italy was headed for a lockdown, and that the Milan airport and train station would be soon closed. There was also news that the first 1 or 2 cases were hospitalized in Lugano. Hence we weren’t sure that there would be a partial lockdown in Lugano, even taxis in Lugano after our rail arrival to get us to our hotels and the congress center, or whether the congress center might be closed by the Lugano authorities. Most immediate and ominous was news that scheduled speakers, and attendees, were cancelling *en masse* because they were fearful of arriving in the middle of an exploding epicenter, and whether they would be allowed entry into Switzerland or transit airports en route, or fearful they would be put into quarantine once they returned to their home countries, or because their institutions had forbidden travel outside of their immediate home areas. What ensued was a series of panicky nonstop discussions, and nonstop phone calls between the Zurich arrival group and Dr. Denning, as we all tried to figure out what to do.

What we eventually worked out was to have some absent speakers give their talks “live” from their home locales via the internet, and others (and this was a necessity because “live” talks were impossible for some, because of global time zone differences) to record their talks and email them, to be projected at the meeting. This meant, we had to find, with essentially no notice, a local audiovisual and computer crew who could cope with needs unanticipated in the meeting planning. In addition, there were innumerable incoming phone, email and fax messages with questions from attendees and speakers about whether the meeting would proceed and what would happen, including absent poster presenters who were trying to arrange colleagues who had already arrived to present the posters of those who now couldn’t or wouldn’t come, and scholarship awardees, from developing countries, who had never travelled internationally before, not to mention into a presumed epidemic area. We also had to cope with suddenly decreased numbers for the ancillary congress events, and food and drinks for meeting breaks that had been booked.

As it turned out, roughly half the scheduled speakers and poster presenters, and half the audience, never made it to Lugano. However, very few scheduled talks were not able to be given, using the remote tools described above, although the original schedule had to be re-arranged for many. Kudos to all the speakers and poster presenters who did make it to the meeting, or cooperated with the alternate plans. Attendees who made it were lavish in their positive evaluations of what the meeting did provide for them. I would nominate, on behalf of my 2 Co-Organizers and myself, that the true heroine of the egress from the chaos would be Derry, and her team, and their improvisation and efforts to go ahead, as closely as possibly could be done, with the original plan.

I thank Cornelia Lass-Florl, Chair of the AAAM9 Scientific Committee (and Chief Editor of the Supplement), and all the Committee. I thank the crew of the Journal of Fungi, whose careful work made this Supplement possible; the Guest Editors (Praveen Juvvadi, Chris Kosmidis, Frederic Lamoth, Gabriele Sass), drawn from the meeting faculty; and the reviewers chosen for these submissions, for all their efforts. I thank the many corporate and foundation sponsors; without their support, this conference would not have been possible. I have special thanks to my 2 fellow Co-Organizers, whose unstinting willingness to take on the many necessary tasks, in the amazing absence of any quarrels among us, since 2003, make these meetings possible. Our founding vision of what we wanted the meetings to achieve has been, in our opinion, realized. We look forward with uncertainty to the 10th Meeting, which had originally been scheduled to take place in Lyon in 2022, as we do not know how the world will look after COVID-19 (follow our website to see what will be planned for the meeting). The format may be modified, if necessary, but the diseases caused by these filamentous fungi have not lessened in importance as a result of the coronavirus, and in fact, these opportunistic pathogens are riding in on the wave of coronavirus infections, in intensive care units and hospitals [4,5,6,7].

## References

[1] Stevens D.A. (2005). Convocation. Med. Mycol..

[2] Clemons K.V., Perlin D., Richardson M. (2012). Preface for Advances against Aspergillosis. Ann. N. Y. Acad. Sci..

[3] Stevens D.A., Melikian G.L. (2011). Aspergillosis in the ‘nonimmunocompromised’ host. Immunol. Invest..

[4] Verweij P.E., Gangneux J.-P., Bassetti M., Brüggemann R.J.M., Cornely O.A., Koehler P., Lass-Flörl C., Van De Veerdonk F.L., Chakrabarti A., Hoenigl M. (2020). Diagnosing COVID-19-associated pulmonary aspergillosis. Lancet Microbe.

[5] Arastehfar A., Carvalho A., Van de Veerdonk F.L., Jenks J.D., Koehler P., Krause R., Cornely O.A., Perlin D.S., Lass-Flörl C., Hoenigl M. (2020). COVID-19 associated pulmonary aspergillosis (CAPA)—From immunology to treatment. J. Fungi.

[6] Thompson G.R., Cornely O.A., Pappas P.G., Patterson T.F., Hoenigl M., Jenks J.D., Clancy C.J., Nguyen M.H. (2020). Invasive Aspergillosis as an Under-recognized Superinfection in COVID-19. Open Forum Infect. Dis..

[7] Koehler P., Cornely O.A., Böttiger B.W., Dusse F., Eichenauer D.A., Fuchs F., Hallek M., Jung N., Klein F., Persigehl T. (2020). COVID-19 associated pulmonary aspergillosis. Mycoses.

